# P01.05. Increase in blood flow associated with traditional chinese medicine therapies

**DOI:** 10.1186/1472-6882-12-S1-P5

**Published:** 2012-06-12

**Authors:** S Lin, T Ross, N Sutherland, G Orenstein

**Affiliations:** 1 University of California, Irvine, Irvine, USA

## Purpose

Many Traditional Chinese Medicine (TCM) therapies are supposed to "move the blood" to enhance health and healing. This pilot study compared the time course of how these therapies increased the flow of blood measured at skin level.

## Methods

The experiments involved a handful of healthy subjects in their 20's-60's. Blood flow was measured as "flux" of cutaneous blood perfusion using a laser Doppler flowmetry instrument (DRT4 from Moor). In each experiment, continuous recording was made to establish a baseline level prior to treatment, and recording was resumed after treatment until baseline level was reached again.

## Results

First we found that needle insertion at various acupuncture points on the back produced an immediate and sharp peak of increased blood flow measured at the treated point which lasted only a few seconds, followed by a second broader peak of 3-4 fold increase that lasted about 5-15 minutes. Cupping on acupoint GB21 produced a similar increase that lasted 15-20 minutes, as did Gua Sha administered on points along the bladder meridian on the back. Similarly, 5 minutes of acupressure massage on the shoulder increased blood flow at GB21 by around 5 fold, diminishing to baseline level within half an hour. In contrast, 5 minute moxibustion treatment on GB21 produced about a 10 fold increase that lasted an hour or two. The longer duration of the effect was also seen in the 4-5 fold increase produced by a 5-minute treatment on PC6 with an over-the-counter herbal remedy commonly used for bruises and sprains ("Tree Head Essence" from Ren Ji Biopharmacological Technology Co., Taiwan).

## Conclusion

In conclusion, we found that the different therapies tested were all very effective in increasing blood flow as measured. Our observation that the treatments involving chemicals lasted significantly longer than the other ones most likely reflects different mechanisms behind their healing effects.

